# The Effect of Potentiostatic Control on the Bioreduction of Hexavalent Chromium Using *Bacillus cereus*

**DOI:** 10.3390/microorganisms14010014

**Published:** 2025-12-20

**Authors:** Huimei Chi, Man Feng

**Affiliations:** 1 State Key Laboratory of Digital Medical Engineering, School of Biological Science & Medical Engineering, Southeast University, Nanjing 211189, China; 2 State Key Laboratory of Palaeontology and Stratigraphy, Nanjing Institute of Geology & Palaeontology, Chinese Academy of Sciences, Nanjing 210008, China

**Keywords:** hexavalent chromium, *Bacillus cereus*, bioelectrochemical system, potentiostatic control, biofilm

## Abstract

Coupling microbial catalysis with electrochemical stimulation offers a promising strategy for heavy metal remediation. This study investigates how potentiostatic control influences the bioreduction of hexavalent chromium (Cr(VI)) by *Bacillus cereus* strain DIF1 in a bioelectrochemical system. Cr(VI) reduction was evaluated under various applied cathodic potentials, and the highest reduction efficiency (91.45%) was achieved at +0.04 V after 24 h. This performance significantly surpassed that of the abiotic control (82.55%) and the open-circuit biotic control (9.25%), indicating that the applied potential enhances microbial Cr(VI) reduction beyond contributions from abiotic processes alone. Cyclic voltammetry (CV) revealed a distinct redox feature at +0.04 V with no corresponding reverse peak, indicating kinetically favored electron transfer during Cr(VI) reduction under this condition. Microscopic imaging confirmed that, under the applied potential, *Bacillus cereus* DIF1 formed filamentous connections, exhibited higher chromium accumulation on bacterial cells than on the surrounding carbon paper electrode, and developed a robust biofilm on the cathode surface. The system maintained consistent Cr(VI) reduction performance over three consecutive cycles, demonstrating good short-term operational reproducibility. These findings highlight the critical role of precise electrochemical control in modulating microbial Cr(VI) reduction and provide mechanistic insights into the interplay between electrode potential and bacterial activity.

## 1. Introduction

Chromium (Cr) is a pervasive heavy metal pollutant resulting from industrial activities such as metal processing, electroplating, leather tanning, mining, and, to a lesser extent, agricultural livestock breeding [1,2,3]. These practices lead to severe contamination of soil and aquatic environments [1]. In the water environment, chromium predominantly exists in two stable oxidation states: the highly toxic and mobile hexavalent chromium (Cr(VI)) and the significantly less toxic and immobile trivalent chromium (Cr(III)) [2].

Cr(VI) poses a critical global concern because of its high solubility and classification as a Group 1 human carcinogen [2]. Its toxicity stems from its strong oxidizing power, which triggers mechanisms like increased oxidative stress, DNA damage, and chromosome breaks [2,4]. Conversely, Cr(III) is relatively harmless to humans, has low solubility, and readily precipitates as Cr(OH)_3_ at neutral pH [2,5]. Therefore, the effective removal and detoxification of Cr(VI) remain a major direction in solving heavy metal pollution problems [1].

To address the environmental risk posed by Cr(VI), efficient and cost-effective remediation strategies are essential [1]. Although conventional methods (e.g., electrochemical treatment and adsorption) are effective, they are often limited by high energy consumption, significant costs, and the generation of secondary chemical sludge [1,6]. Bioremediation, utilizing microorganisms to detoxify pollutants, presents a promising, sustainable and eco-friendly alternative [6,7,8]. The core of chromium bioremediation is the microbial reduction of Cr(VI) to Cr(III) [7,9,10]. This detoxification process is achieved by various microbial strains, including bacteria and fungi [2,7,8,10,11,12,13,14].

Microbial Cr(VI) reduction is a complex process involving multiple mechanisms where reduction occurs both intracellularly and extracellularly [11,12,14,15]. The cellular defense mechanisms against Cr(VI) toxicity include the synthesis of stress-relieving compounds and the use of efflux pumps [4,14,16]. The resulting Cr(III) product is typically sequestered through adsorption or precipitation on the cell surface or released as soluble organo-Cr(III) complexes [10,12,13].

To improve overall remediation efficiency, researchers have focused on enhancement strategies such as environmental tuning, where the optimization of physicochemical parameters like pH and temperature is crucial [8,10,11]. The presence of specific ions (e.g., Ca^2+^, SO_4_^2−^, or Cu(II)) can also positively influence reduction rates and microbial tolerance [14,16]. Furthermore, Bioelectrochemical Systems (BESs) such as Microbial Fuel Cells (MFCs) offer a superior approach by enabling simultaneous cathodic Cr(VI) reduction and energy recovery [1,17,18]. In these systems, electrochemically active bacteria (EAB) in the biocathode (e.g., *Shewanella oneidensis*, *Bacillus* sp.) utilize electrons for the reduction reaction [5,19]. Finally, strategies for accelerating electron transfer can enhance MFC performance through the use of electron shuttle mediators (e.g., humic acid, Cu(II) ions, riboflavin) to improve extracellular electron transfer (EET) [15,19,20,21].

Although microbial reduction offers high potential, challenges remain in achieving robust and efficient performance under key real-world conditions. These challenges include operating under high pollutant concentrations, mixed pollution scenarios (i.e., multiple heavy metals present simultaneously) [22,23], and dynamic environmental conditions, such as the redox alternations frequently found in soil groundwater systems [24]. Furthermore, optimizing the use of highly tolerant or engineered strains in practical, large-scale systems, such as MFCs, still requires detailed mechanistic understanding and improved operational strategies [25,26].

Despite recent progress, most Cr(VI) reducing biocathodes rely on model electroactive bacteria such as *Shewanella* or *Pseudomonas*, with limited attention given to *Bacillus cereus* strains under defined electrochemical control [5,19]. Although potentiostatic strategies have been explored in other bioelectrochemical systems, their specific impact on Cr(VI) reduction by non-model *Bacillus* isolates and the extent to which microbial activity truly enhances performance beyond abiotic electrochemical effects remain unclear [18,25]. This study addresses these gaps by evaluating a newly isolated *Bacillus cereus* strain DIF1 under multiple applied potentials, directly comparing biotic and abiotic controls to quantify the microbial contribution and to identify an optimal potential for enhanced Cr(VI) bioreduction.

In this study, we introduce our self-isolated, highly efficient chromium-reducing bacterial strain, *Bacillus cereus* strain DIF1. We utilize this strain as a biocatalyst for Cr(VI) reduction within the cathode of a Microbial Fuel Cell (MFC). Specifically, we investigate the effect of applying different external voltages to the MFC system. This potentiostatic control technique is employed as an effective operational strategy to optimize and accelerate the microbial electron transfer processes, thereby enhancing the overall Cr(VI) reduction efficiency [27]. Our findings characterize the performance of this novel biocatalyst and provide a proven, effective operational strategy for improving MFC-based Cr(VI) remediation, thus offering a novel approach and methodology for addressing environmental chromium contamination.

## 2. Materials and Methods

### 2.1. Bacillus Cultivation and Microbial Fuel Cell Configuration

The bacterial strain *Bacillus cereus* DIF1 (deposited in CCTCC as M2018274) was isolated and cultured in our lab. Prior to inoculation, the strain was precultivated and enriched in liquid LB broth in an incubator at 30 °C with shaking at 120 rpm for 24 h. Bacteria were harvested by centrifugation at 5000 rpm for 10 min and then re-suspended in DM medium. Two-chambered electrochemical fuel cells were designed and fabricated from glass, with each chamber having a working volume of 150 mL. Nafion-117 (DuPont, Wilmington, DE, USA) served as the proton exchange membrane (PEM) to separate the anode and cathode. Carbon paper (2 cm × 5 cm; effective area 2 cm × 2.5 cm) was used as the electrode material for both the anode and cathode. A 1000 ohm resistor was used as the external load. All reactors were placed in the incubator in the dark and the temperature was set at 25 °C.

### 2.2. Cr(VI) Reduction in MFC Cathode

DM medium (containing 2.5 g/L ${NaHCO}_{3}$, 0.08 g/L $Ca{Cl}_{2}$, 1.0 g/L ${NH}_{4}$Cl, 0.2 g/L Mg${Cl}_{2}$, 10 g/L NaCl, 7.2 g/L HEPES, 0.5 g/L Yeast, 1.76 g/L sodium lactate, the pH was adjusted to 7.2 with NaOH or HCl) and *Bacillus cereus* strain DIF1 were present in both chambers. Chromium(VI) was introduced into the cathode by adding 2 mmol/L Cr(VI) (prepared by dissolving Na_2_CrO_4_ in deionized water). Meanwhile, abiotic cathodes in the MFCs served as the control groups. Electrochemical performance was assessed based on the rate of Cr(VI) reduction. We conducted a series of Cr(VI) reduction experiments across a wide potential range (−0.8 V to 0.8 V). Three constant potentials (+0.04 V, +0.002 V, and −0.04 V) were selected from this range for the electroreduction of Cr(VI) by the strain, as they demonstrated the most efficient Cr(VI) reduction. Cr(VI) reduction was monitored at defined time intervals by detecting the optical density (OD) using a UV-VIS spectrophotometer (SHIMADZU, UV-2600, Kyoto, Japan), following the standard Diphenylcarbazide method. All absorbance measurements were carried out at 540 nm, the well-established wavelength of maximum absorption for the Cr(VI) diphenylcarbazide (DPC) complex in acidic medium. A six-point external calibration curve was prepared using freshly made Cr(VI) standards (from Na_2_CrO_4_) in the same DM medium used in the experiments, covering a concentration range of 0 to 2.0 mmol/L. Based on triplicate measurements, the calibration equation was: A_540_ = 0.482XC_Cr(VI)_ + 0.008(R^2^ = 0.9992). The method exhibited a linear range of 0.02–2.0 mmol/L.

### 2.3. Scanning Electron Microscopy (SEM)

A Zeiss Ultra Plus Field Emission Scanning Electron Microscope (SEM) (Oberkochen, Germany) was used to observe the morphological characteristics of the bacteria on the carbon paper anodes. The samples were pretreated as follows: First, small pieces of carbon paper from the anodes were fixed in 2.5% glutaraldehyde solution for 2 h. They were then washed three times with 0.2 mmol PBS (PH 7.2), and subsequently washed three times with deionized water. Next, the samples were successively dehydrated for 5 min each in a series of t-butanol/ethanol mixtures (50%, 70%, and 90% t-butanol). Finally, they were dehydrated three more times using 100% t-butanol for 5 min per wash, followed by overnight drying. Additionally, the elemental composition was characterized via Energy Dispersive Spectroscopy (EDS).

### 2.4. Transmission Electron Microscope (TEM)

Bacterial precipitates of reaction solution cultured were collected by centrifugation (10,000× *g*, 10 min) at 4 °C. The fixative was prepared as 4% Glutaraldehyde dissolved in 0.1 M Phosphate-Buffered Saline (PBS, pH 7.0). The bacterial pellet was gently resuspended in the fixative, or an equal volume of double-concentration fixative was added directly to the culture medium. Fixation was carried out at 4 °C for 2 h to overnight. Residual glutaraldehyde was removed by washing the sample 3 times (15 min each wash) with the same concentration of buffer (without glutaraldehyde). The sample was then collected using a copper mesh and air-dried. The copper mesh containing the sample was subsequently observed using a Transmission Electron Microscope (TEM, JEM-2100, Tokyo, Japan).

### 2.5. Bioelectrochemical Experiments

The bioelectrochemical measurements were conducted using a three-electrode configuration linked to a CHI600E electrochemical workstation (Chenhua, Shanghai, China). An Ag/AgCl electrode (R0303, Tianjin, China) served as the reference electrode, and the carbon paper served as both the working electrode and the counter electrode. Cyclic Voltammetry (CV) was conducted to study the electrochemical behavior, with a scan rate of 10 mV/s ranging from −1 V to 0.6 V. Differential Pulse Voltammetry (DPV) was adopted for higher-sensitivity measurements. Amperometric I–t curves were performed at a constant potential of −500 mV for the potentiostatically controlled cathodes.

## 3. Results

### 3.1. Effects of Applied Voltage on Cr(VI) Reduction and Bacillus cereus DIF1 Growth

**Figure 1:**
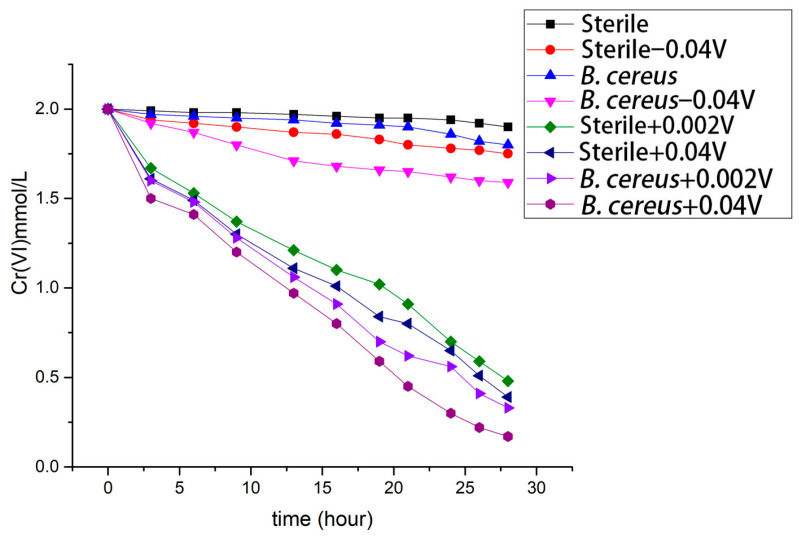
Bioreduction of Cr(VI) by *Bacillus cereus* strain DIF1 under applied potential.

The application of positive potentials significantly enhanced Cr(VI) reduction in both biotic and abiotic systems. In sterile (abiotic) controls, Cr(VI) concentrations decreased substantially under +0.002 V and +0.04 V (Table 1, Figure 1), indicating that electrochemical reduction contributed to Cr(VI) removal even in the absence of bacteria. However, the most effective Cr(VI) reduction was observed in biotic systems where *Bacillus cereus* strain DIF1 was present alongside an applied potential. Among all tested conditions, the combination of DIF1 with +0.04 V achieved the lowest residual Cr(VI) concentration (0.17 mmol/L), which was significantly lower than that of all other groups (Tukey’s HSD, *p* < 0.05).

A negative potential (−0.04 V) provided a slight, albeit statistically non-significant, enhancement in Cr(VI) reduction compared to the DIF1 control without voltage (*p* > 0.05). Within the sterile groups, no significant difference was observed between +0.002 V and +0.04 V; however, under biotic conditions, +0.04 V (*Bacillus cereus* + 0.04 V) was significantly more effective than sterile + 0.04 V, highlighting the synergistic interaction between the bacterial strain and the applied potential.

**Figure 2:**
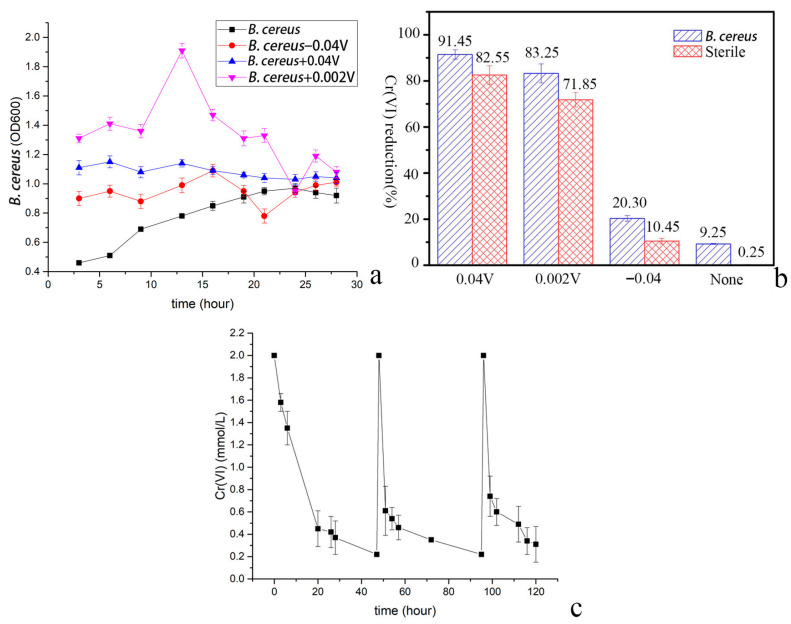
Cr(VI) reduction performance under various applied potentials. (**a**) the growth curve of *Bacillus cereus* DIF1 under different applied potentials, (**b**) biotic and abiotic comparison of Cr(VI) reduction at applied potentials, (**c**) Cr(VI) concentration in the biocathode MFC in the presence of *Bacillus cereus* + 0.04.

As shown in Table 2 and Figure 2a, the OD_600_ of *Bacillus cereus* DIF1 at +0.002 V (1.47 ± 0.03) was significantly higher than under all other voltage conditions (*p* < 0.05), suggesting that a weak positive potential is most conducive to cell proliferation. Both −0.04 V and +0.04 V significantly promoted growth relative to the control (0 V), but did not differ significantly from each other, indicating comparable growth stimulating effects of these two potentials. The dissociation between maximal growth (+0.002 V) and maximal Cr(VI) reduction (+0.04 V) implies that reduction efficiency is not solely dependent on biomass accumulation, but likely involves electroactive metabolism or voltage regulated enzymatic pathways. Notably, while +0.04 V yielded the highest Cr(VI) reduction efficiency (91.45%), the optimal condition for bacterial growth was +0.002 V, see Figure 2b. The three-cycle reduction experiment demonstrated the stability of the reduction process, see Figure 2c and Table 3.

### 3.2. Cyclic Voltammetry Analysis Under Different Applied Potentials

**Figure 3:**
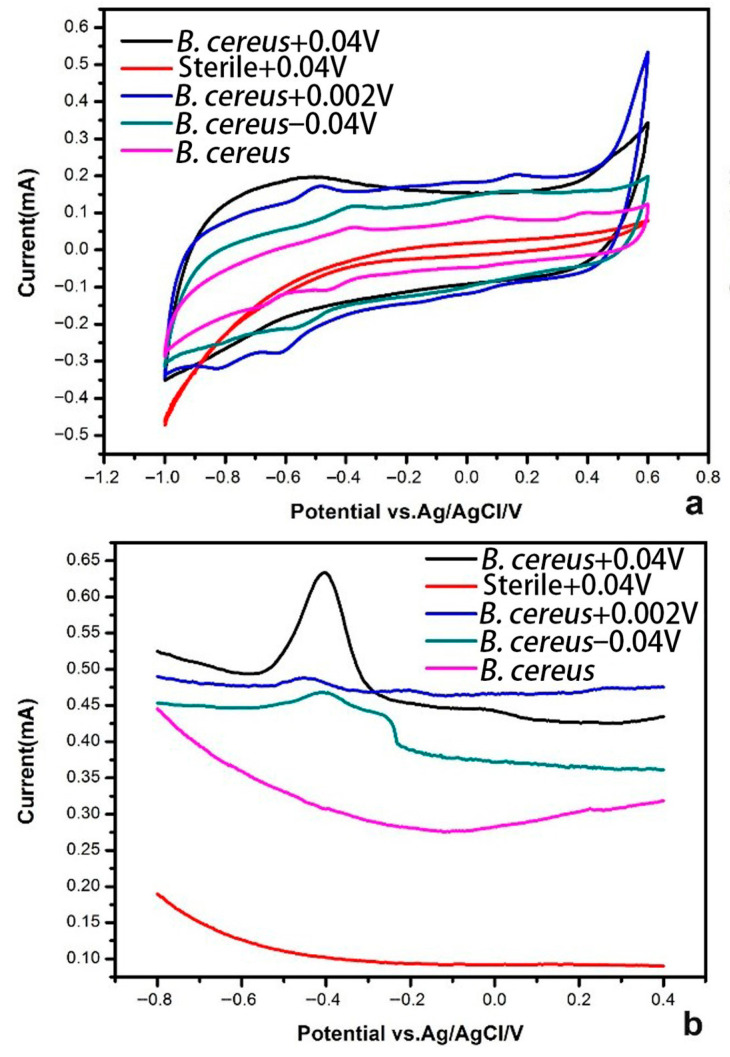
Cyclic Voltammetry at the potential range from −0.8 to 0.6 V under different conditions. (**a**) Cyclic Voltammetry (CV), (**b**) Differential Pulse Voltammetry (DPV).

Cyclic voltammetry (CV) measurements conducted after a 2-day incubation revealed marked differences between biotic (with *Bacillus cereus* DIF1) and abiotic (sterile) systems, particularly under an applied potential of +0.04 V. In the biotic system polarized at +0.04 V, a single, well-defined oxidation peak was observed at −0.51 V with a current of 0.2 mA, see Figure 3a. Notably, no corresponding reduction peak appeared during the reverse scan, suggesting an irreversible electrochemical process likely associated with bacterial-mediated Cr(VI) reduction or redox-active metabolite oxidation. In contrast, no redox peaks were detected in the sterile control under the same +0.04 V condition, confirming that the observed electroactivity originated from biological activity rather than abiotic reactions.

Biotic systems under non-optimal potentials, namely +0.002 V, −0.04 V and no applied potential (0 V), exhibited more complex voltammetric profiles, each showing multiple pairs of redox peaks within the range of −0.38 V to −0.48 V. This multi-peak behavior may reflect heterogeneous electron transfer pathways or the involvement of various redox active cellular components under suboptimal electrochemical conditions.

To further evaluate early stage electrocatalytic activity, Differential Pulse Voltammetry (DPV) was performed after 4 h of incubation, see Figure 3b. The results demonstrated that the presence of DIF1 significantly enhanced the system’s electrical response, and that application of an appropriate potential (particularly +0.04 V) markedly improved the catalytic activity of the bacterial strain. These findings corroborate the CV data and support the conclusion that potentiostatic control at +0.04 V optimizes the electroactive interaction between DIF1 and the electrode, thereby facilitating efficient Cr(VI) reduction.

### 3.3. Potentiostatically Controlled Amperometric I–t Curve Analysis

Amperometric current–time (I–t) curves were recorded at a fixed potential of −500 mV after a 4-h conditioning period under various applied potentials. Although the detection potential was identical across all systems, subtle but discernible differences in current response were observed, indicating that the preceding conditioning potential significantly influenced the subsequent electrochemical behavior of the system.

**Figure 4:**
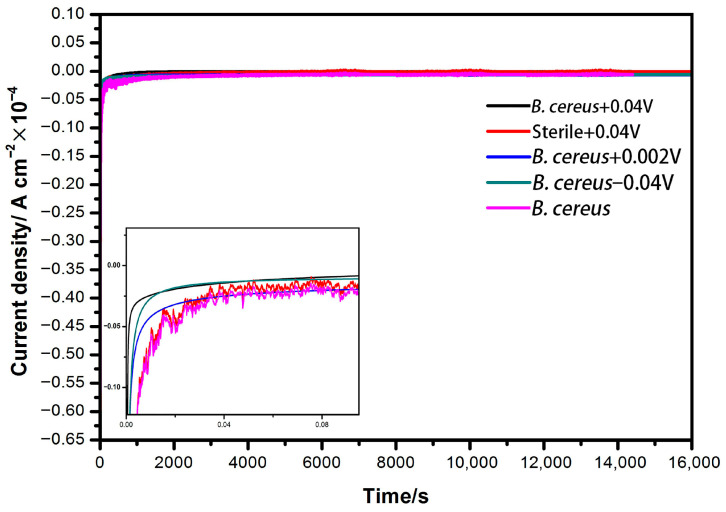
Amperometric I–t curves at −500 mV for the potentiostatically controlled.

Among the biotic treatments, the system pre-conditioned with *Bacillus cereus* DIF1 at +0.04 V exhibited the fastest initial current rise, reflecting rapid electron transfer kinetics. In contrast, the system pre-conditioned at −0.04 V showed the highest initial current magnitude, but this response decayed rapidly over time, eventually ranking third in sustained current output (Figure 4).

By comparison, the abiotic control pre-conditioned at +0.04 V and the biotic system with no applied conditioning potential (0 V) displayed only minor current fluctuations, with negligible stable faradaic response. These results demonstrate that the electrochemical “memory” or activation state of the bacterial cells is modulated by the conditioning potential, and that +0.04 V optimally primes DIF1 for efficient electron transfer, consistent with its superior Cr(VI) reduction performance observed in batch experiments.

### 3.4. Microscopic and Elemental Evidence of Bacterial Involvement in Cr(VI) Reduction

**Figure 5:**
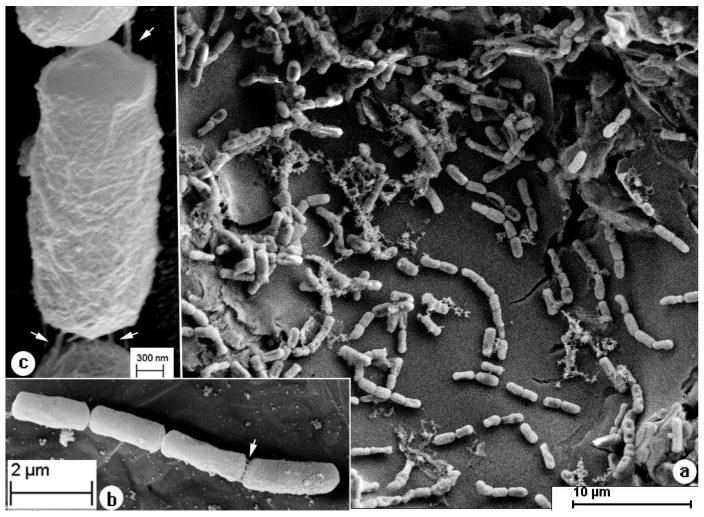
SEM image of *Bacillus cereus* DIF1 in the biocathode MFC in the presence of *Bacillus cereus* + 0.04. (**a**) SEM image of *Bacillus cereus* DIF1 in the biocathode MFC, (**b**) enlargement of a part of (**a**), (**c**) enlargement of a part of (**b**). Arrows in (**b**,**c**) indicate filaments between DIF1.

Scanning electron microscopy (SEM) observations of *Bacillus cereus* DIF1 during voltage-driven Cr(VI) reduction revealed that the cells were interconnected, forming filamentous structures between adjacent bacteria (Figure 5a–c). This morphological feature strongly suggests the formation of a biofilm-like architecture on the cathode surface, which may facilitate electron transfer and enhance metal reduction efficiency.

**Figure 6:**
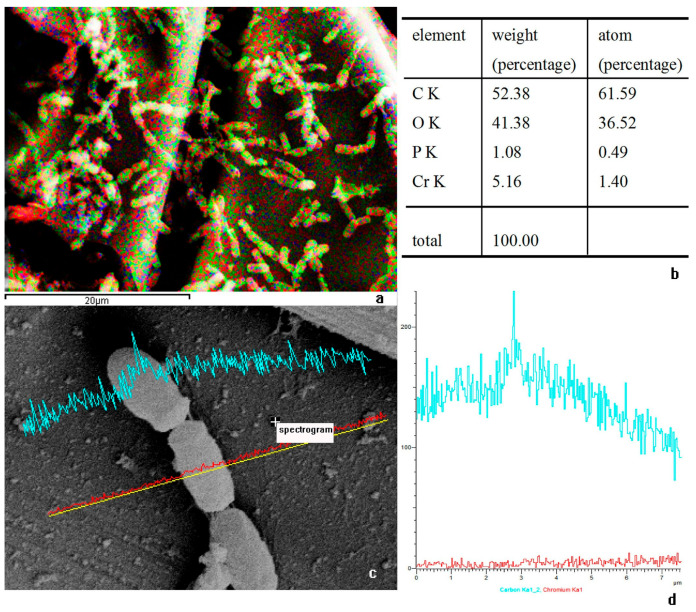
Image of energy dispersive spectroscopy (EDS) by SEM, (**a**) Multi-element composite image, red represents carbon (C); green represents oxygen (O); yellow represents phosphorus (P); blue represents Chromium (Cr). (**b**) percentage of multi-element, CK: K-shell X-ray emission from carbon, OK: K-shell X-ray emission from oxygen; PK: K-shell X-ray emission from phosphorus; CrK: K-shell X-ray emission from chromium. (**c**) spectrogram of Chromium, blue represents Chromium (Cr). (**d**) Chromium abundance curve, blue represents Chromium (Cr).

Energy dispersive X-ray spectroscopy (EDS) analysis of the bacterial region showed the following elemental composition: Carbon (C): 61.59%, Oxygen (O): 36.52%, Chromium (Cr): 1.40% and Phosphorus (P): 0.49% (Figure 6b). Notably, chromium was the third most abundant element after C and O, indicating substantial Cr accumulation associated with the bacterial biomass. Elemental mapping further demonstrated that Cr was uniformly distributed across the bacterial surface (Figure 6c), and its concentration was markedly higher on the bacteria than on the surrounding carbon paper electrode (Figure 6d). This spatial enrichment of Cr on microbial cells rather than on the bare electrode provides indirect evidence that the bacteria play a central role in Cr(VI) immobilization and reduction, particularly when coupled with an appropriate external potential.

**Figure 7:**
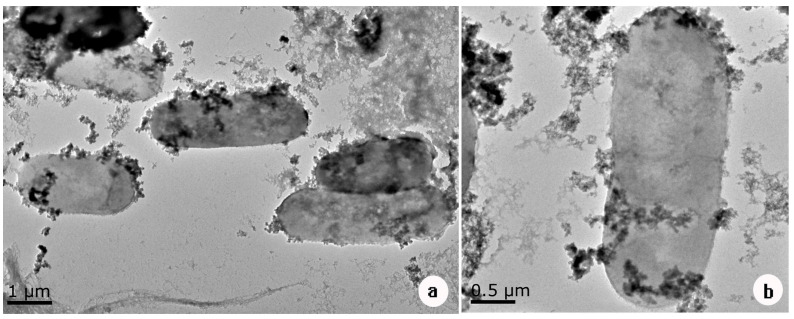
TEM image of Cr(VI) reduction by *Bacillus cereus* DIF1. (**a**) TEM images of Cr(VI) reduction in a group of bacteria, (**b**) enlarged image of Cr(VI) reduction in a bacteria.

Transmission electron microscopy (TEM) images corroborated these findings, revealing a dense accumulation of electron-dense granular material surrounding the DIF1 cells (Figure 7a,b). While minor particle deposition was also observed on the carbon paper, the vast majority of granules were localized in close proximity to the bacterial envelopes. These granules are likely composed of reduced chromium species (e.g., Cr(III) precipitates), and their preferential association with bacterial cells further supports the conclusion that DIF1 serves as the primary catalytic agent for Cr(VI) reduction under potentiostatic conditions.

## 4. Discussion

The increase in Cr(VI) reduction from 9.25% (biotic, no potential) to 91.45% (biotic, +0.04 V) demonstrates a clear synergistic effect between *Bacillus cereus* DIF1 and the applied electrical potential. The abiotic system at +0.04 V achieved 82.55% reduction, indicating significant electrochemical reduction on the carbon electrode; however, the additional 8.9% reduction in the biotic system confirms that DIF1 contributes meaningfully under potentiostatic control [28]. This is consistent with bioelectrochemical principles where the cathode supplies electrons for microbial reduction processes [29,30]. The applied potential likely enhances extracellular electron transfer (EET) to Cr(VI), similar to how artificial mediators such as neutral red or biochar improve reduction rates [31,32,33]. Improved Cr(VI) reduction in the second and third cycles suggests a time-dependent process involving biofilm development. *B. cereus* is known to form heterogeneous biofilms [34], and the progressive performance aligns with the time required for cell attachment, Extracellular polymeric substance (EPS) production, and biomass accumulation on the cathode surface [34,35]. The resulting biofilm may stabilize EET pathways and support sustained activity.

EPSs, rich in functional groups, play a key role in Cr(VI) biosorption [36,37], localizing the toxic species near cell-associated reductases or EET components [38]. Repeated exposure to Cr(VI) over cycles may also promote physiological adaptation, potentially involving the upregulation of resistance or reduction mechanisms [37,39], contributing to stable performance.

Electrochemical data confirm the essential role of DIF1. No redox peaks were detected in the abiotic CV at +0.04 V (Figure 3a), whereas the biotic system showed a distinct oxidation peak at −0.51 V, indicating biological electroactivity. DPV results further show that an appropriate potential enhances bacterial catalytic response (Figure 3b), supporting the concept of electrochemically assisted bioremediation [29,40,41]. The cathode likely provides the reducing power required for microbial EET [30]. CV profiles varied with the applied potential. Systems at non-optimal potentials (+0.002 V, −0.04 V, 0 V) exhibited multiple redox peak pairs between −0.38 V and −0.48 V, possibly reflecting the involvement of various redox-active species or multi-step electron transfer processes [32,42]. In contrast, the +0.04 V system displayed only one irreversible oxidation peak with no reverse reduction peak, suggesting a direct and efficient Cr(VI) reduction pathway. The oxidation peak at −0.51 V likely corresponds to the re-oxidation of Cr(III) precipitates (e.g., CrOOH) formed during reduction [32], and the absence of a cathodic peak indicates the stability of the reduced product under experimental conditions [28,43]. Differences in CV signatures across potentials may reflect variations in the location or mechanism of Cr(VI) reduction, which can affect electron transfer kinetics [31].

Amperometric I–t curves recorded at −500 mV revealed that the conditioning potential influences the subsequent electron transfer behavior. The system preconditioned at +0.04 V showed the fastest current rise, consistent with its superior Cr(VI) reduction and CV profile, suggesting this potential optimizes the interface for electron acceptance [30,31]. In contrast, the system preconditioned at −0.04 V exhibited a high initial current that rapidly decayed, possibly due to transient abiotic contributions followed by passivation or inhibition [28,44]. The minimal responses in the abiotic control and the 0 V biotic system indicate limited faradaic activity without an applied potential [40,45], confirming that the prior electrochemical environment shapes the biocathode’s performance.

Microscopic and elemental analyses provide direct evidence of bacterial involvement. SEM images show interconnected DIF1 cells forming filamentous structures on the cathode (Figure 5a–c), indicative of biofilm formation. EDS detected Cr(1.40%) as the third most abundant element on bacterial surfaces significantly higher than on the surrounding carbon paper (Figure 6b–d) with uniform distribution suggesting a close association between cells and chromium [43]. The presence of phosphorus (0.49%) may relate to cellular components or P-containing EPS [43]. TEM images reveal granular material predominantly localized around bacterial cells (Figure 7a,b), consistent with Cr(III) precipitates such as CrOOH [32]. The preferential accumulation of these granules near bacteria rather than on the bare electrode confirms that DIF1 is the primary site of Cr(VI) reduction. This supports an extracellular mechanism where Cr(VI) is adsorbed and reduced at the cell surface, followed by in situ precipitation of insoluble Cr(III) [37,38,43,46,47], enabling effective detoxification [36,37,40,47]. Together, these results demonstrate that coupling *B. cereus* DIF1 with a +0.04 V potential creates a robust and efficient system for Cr(VI) remediation, integrating microbial activity, the electrochemical driving force, and biofilm mediated reduction. Notably, despite repeated Cr(VI) reduction cycles, no significant decline in performance was observed, suggesting that extracellular Cr(III) precipitates did not cause substantial passivation of the biocathode—likely due to their localized deposition on bacterial cells rather than on the electrode surface itself [32,37,43,48].

Despite the enhanced Cr(VI) reduction observed under potentiostatic control, several experimental conditions limit the environmental applicability of this approach. The study was conducted in a synthetic medium with a fixed Cr(VI) concentration, constant pH, and the absence of competing ions or organic matter conditions that differ markedly from those found in real wastewater or contaminated groundwater [28,40]. The presence of co-contaminants (e.g., nitrate, sulfate, or other metals) could either inhibit microbial activity or interfere with electron transfer [37,44,49]. Furthermore, the use of a pure culture of *B. cereus* DIF1 oversimplifies natural microbial communities, which may exhibit different reduction capacities due to interspecies interactions [39,45]. Finally, the reliance on a three-electrode system with precise external potential control is technically demanding and energy-intensive, posing significant barriers to large-scale implementation. Practical deployment would require low-cost, self-sustaining bioelectrochemical configurations (e.g., microbial fuel cells) operating without potentiostats [29,41]. Nevertheless, the demonstrated synergy between mild electrochemical stimulation (+0.04 V) and *B. cereus*-mediated Cr(VI) reduction offers a promising foundation for developing energy-efficient, biohybrid systems tailored for real-world chromium laden wastewater treatment.

## 5. Conclusions

This study demonstrates the efficacy of a bioelectrochemical system integrating *Bacillus cereus* strain DIF1 for Cr(VI) reduction. The results identified +0.04 V as the optimal applied potential, achieving 91.45% Cr(VI) reduction after 24 h significantly higher than the 82.55% reduction observed in the abiotic control under the same potential and far exceeding the performance of biotic systems without electrochemical stimulation. Electrochemical analyses indicated that +0.04 V enhances electron transfer activity compared to other tested potentials. Microscopic observations confirmed robust biofilm formation on the cathode surface under this condition. These findings show that the combination of *Bacillus cereus* DIF1 with precise potentiostatic control significantly improves Cr(VI) reduction efficiency, offering a promising approach for the bioelectrochemical treatment of chromium contaminated environments.

## Figures and Tables

**Table 1:** Bioreduction of Cr(VI) by *Bacillus cereus* strain DIF1 under applied potential.

	Sterile _a_	Sterile − 0.04 V _a_	*B. cereus*_a_	*B. cereus* − 0.04 V _b_	Sterile + 0.002 V _c_	Sterile + 0.04 V _c_	*B. cereus* + 0.002 V _c_	*B. cereus* + 0.04 V _d_
time	Cr(VI)	Cr(VI)	Cr(VI)	Cr(VI)	Cr(VI)	Cr(VI)	Cr(VI)	Cr(VI)
hour	mmol/L	mmol/L	mmol/L	mmol/L	mmol/L	mmol/L	mmol/L	mmol/L
0	2.00 ± 0	2.00 ± 0	2.00 ± 0	2.00 ± 0	2.00 ± 0	2.00 ± 0	2.00 ± 0	2 ± 0
3	1.99 ± 0.07	1.94 ± 0.01	1.97 ± 0	1.92 ± 0	1.67 ± 0.17	1.61 ± 0.13	1.60 ± 0.22	1.50 ± 0.05
6	1.98 ± 0.02	1.92 ± 0.06	1.96 ± 0.09	1.87 ± 0.10	1.53 ± 0.13	1.49 ± 0.10	1.48 ± 0.17	1.41 ± 0.11
9	1.98 ± 0.15	1.90 ± 0.10	1.95 ± 0.13	1.80 ± 0.09	1.37 ± 0.09	1.3 ± 0.07	1.28 ± 0.09	1.20 ± 0.09
13	1.97 ± 0.09	1.87 ± 0.07	1.94 ± 0.20	1.71 ± 0.12	1.21 ± 0.11	1.11 ± 0.04	1.06 ± 0.15	0.97 ± 0.1
16	1.96 ± 0.06	1.86 ± 0.10	1.92 ± 0	1.68 ± 0	1.10 ± 0.09	1.01 ± 0.05	0.91 ± 0.14	0.80 ± 0.05
19	1.95 ± 0.08	1.83 ± 0.11	1.91 ± 0.12	1.66 ± 0.14	1.02 ± 0.07	0.84 ± 0.03	0.7 ± 0.14	0.59 ± 0.17
21	1.95 ± 0.04	1.80 ± 0.09	1.90 ± 0.13	1.65 ± 0.12	0.91 ± 0.06	0.8 ± 0.06	0.62 ± 0.13	0.45 ± 0.09
24	1.94 ± 0.06	1.78 ± 0.08	1.86 ± 0.11	1.62 ± 0.06	0.7 ± 0.05	0.65 ± 0.09	0.56 ± 0.11	0.30 ± 0.07
26	1.92 ± 0.07	1.77 ± 0.1	1.82 ± 0.12	1.60 ± 0.10	0.59 ± 0.08	0.51 ± 0.07	0.41 ± 0.1	0.22 ± 0.05
28	1.90 ± 0.02	1.75 ± 0.05	1.80 ± 0.13	1.59 ± 0.12	0.48 ± 0.07	0.39 ± 0.03	0.33 ± 0.05	0.17 ± 0.04

Significant differences (*n* = 3, Alpha = 0.05, Tukey’s HSD = 0.197). Using Compact Letter Display (CLD), assign letters (where the same letter indicates no significant difference) to the means, sorted from highest to lowest. Groups sharing the letter “a” showed no significant difference. *B. cereus* − 0.04 V alone was assigned “b”. Groups sharing the letter “c” showed no significant difference. *B. cereus* + 0.04 V was significantly lower than the groups sharing “c” (“d”).

**Table 2:** Growth curve of *Bacillus cereus* DIF1 under applied voltage.

Time	*Bacillus cereus*_c_	*Bacillus cereus* − 0.04 V _b_	*Bacillus cereus* + 0.04 V _b_	*Bacillus cereus* + 0.002 V _a_
hour	OD_600_	OD_600_	OD_600_	OD_600_
3	0.46 ± 0.01	0.9 ± 0.01	1.11 ± 0.05	1.31 ± 0.02
6	0.51 ± 0.01	0.95 ± 0.02	1.15 ± 0.04	1.41 ± 0.04
9	0.69 ± 0.01	0.88 ± 0.01	1.08 ± 0.04	1.36 ± 0.04
13	0.78 ± 0.01	0.99 ± 0.02	1.14 ± 0.03	1.91 ± 0.05
16	0.85 ± 0.03	1.09 ± 0.01	1.09 ± 0.03	1.47 ± 0.03
19	0.91 ± 0.04	0.95 ± 0.03	1.06 ± 0.02	1.31 ± 0.05
21	0.95 ± 0.03	0.78 ± 0.02	1.04 ± 0.03	1.33 ± 0.05
24	0.97 ± 0.03	0.94 ± 0.03	1.03 ± 0.03	0.95 ± 0.03

Significant differences (Tukey HSD, Alpha = 0.05, t = 16 h, *n* = 3). Groups labeled with the same letter are statistically indistinguishable (*p* > 0.05).

**Table 3:** Cr(VI) reduction profile of *Bacillus cereus* DIF during three cycles at an applied potential of +0.04 V.

Cycle 1 _a_	Time (hour)	0	3	6	20	28	47
	Cr(VI) (mmol/L)	2.00 ± 0	1.58 ± 0.08	1.35 ± 0.15	0.45 ± 0.16	0.37 ± 0.15	0.22 ± 0
Cycle 2 _a_	Time (hour)	48	51	54	57	72	95
	Cr(VI) (mmol/L)	2.00 ± 0	0.61 ± 0.22	0.54 ± 0.10	0.46 ± 0.11	0.35 ± 0	0.22 ± 0
Cycle 3 _a_	Time (hour)	96	99	102	112	116	120
	Cr(VI) (mmol/L)	2.00 ± 0	0.74 ± 0.18	0.60 ± 0.12	0.49 ± 0.16	0.34 ± 0.12	0.31 ± 0.16

Tukey’s HSD test (Alpha = 0.05) was applied to compare the final Cr(VI) concentrations across three consecutive reduction cycles under *Bacillus cereus* + 0.04 V conditions (*n* = 3). The mean residual Cr(VI) levels were 0.22, 0.22 and 0.31 mmol/L for Cycles 1, 2, and 3, respectively. No statistically significant differences were observed among the cycles (HSD = 0.231, *p* > 0.05), indicating good reproducibility of the Cr(VI) reduction performance over repeated batches. “a” indicates no significant difference among the three cycles.

## Data Availability

The original contributions presented in this study are included in the article. Further inquiries can be directed to the corresponding author.
